# Lassa virus-like particles displaying all major immunological determinants as a vaccine candidate for Lassa hemorrhagic fever

**DOI:** 10.1186/1743-422X-7-279

**Published:** 2010-10-20

**Authors:** Luis M Branco, Jessica N Grove, Frederick J Geske, Matt L Boisen, Ivana J Muncy, Susan A Magliato, Lee A Henderson, Randal J Schoepp, Kathleen A Cashman, Lisa E Hensley, Robert F Garry

**Affiliations:** 1 Tulane University Health Sciences Center, New Orleans, LA, USA; 2 Autoimmune Technologies, LLC, New Orleans, LA, USA; 3 Corgenix Medical Corporation, Broomfield, CO, USA; 4 Tulane University Department of Pathology, New Orleans, LA, USA; 5 Vybion, Inc., Ithaca, NY, USA; 6 Applied Diagnostics Branch, Diagnostic Systems Division, U.S. Army Medical Research Institute of Infectious Diseases, Fort Detrick, MD, USA; 7 Viral Therapeutics Branch, Virology Division, U.S. Army Medical Research Institute of Infectious Diseases Diagnostic Systems Division, Fort Detrick, MD, USA

## Abstract

**Background:**

Lassa fever is a neglected tropical disease with significant impact on the health care system, society, and economy of Western and Central African nations where it is endemic. Treatment of acute Lassa fever infections has successfully utilized intravenous administration of ribavirin, a nucleotide analogue drug, but this is not an approved use; efficacy of oral administration has not been demonstrated. To date, several potential new vaccine platforms have been explored, but none have progressed toward clinical trials and commercialization. Therefore, the development of a robust vaccine platform that could be generated in sufficient quantities and at a low cost per dose could herald a subcontinent-wide vaccination program. This would move Lassa endemic areas toward the control and reduction of major outbreaks and endemic infections. To this end, we have employed efficient mammalian expression systems to generate a Lassa virus (LASV)-like particle (VLP)-based modular vaccine platform.

**Results:**

A mammalian expression system that generated large quantities of LASV VLP in human cells at small scale settings was developed. These VLP contained the major immunological determinants of the virus: glycoprotein complex, nucleoprotein, and Z matrix protein, with known post-translational modifications. The viral proteins packaged into LASV VLP were characterized, including glycosylation profiles of glycoprotein subunits GP1 and GP2, and structural compartmentalization of each polypeptide. The host cell protein component of LASV VLP was also partially analyzed, namely glycoprotein incorporation, though the identity of these proteins remain unknown. All combinations of LASV Z, GPC, and NP proteins that generated VLP did not incorporate host cell ribosomes, a known component of native arenaviral particles, despite detection of small RNA species packaged into pseudoparticles. Although VLP did not contain the same host cell components as the native virion, electron microscopy analysis demonstrated that LASV VLP appeared structurally similar to native virions, with pleiomorphic distribution in size and shape. LASV VLP that displayed GPC or GPC+NP were immunogenic in mice, and generated a significant IgG response to individual viral proteins over the course of three immunizations, in the absence of adjuvants. Furthermore, sera from convalescent Lassa fever patients recognized VLP in ELISA format, thus affirming the presence of native epitopes displayed by the recombinant pseudoparticles.

**Conclusions:**

These results established that modular LASV VLP can be generated displaying high levels of immunogenic viral proteins, and that small laboratory scale mammalian expression systems are capable of producing multi-milligram quantities of pseudoparticles. These VLP are structurally and morphologically similar to native LASV virions, but lack replicative functions, and thus can be safely generated in low biosafety level settings. LASV VLP were immunogenic in mice in the absence of adjuvants, with mature IgG responses developing within a few weeks after the first immunization. These studies highlight the relevance of a VLP platform for designing an optimal vaccine candidate against Lassa hemorrhagic fever, and warrant further investigation in lethal challenge animal models to establish their protective potential.

## Background

Lassa virus, a member of the *Arenaviridae *family, is the etiologic agent of Lassa fever, which is an acute and often fatal illness endemic to West Africa. There are an estimated 300,000 - 500,000 cases of Lassa fever each year [1-3], with a mortality rate of 15%-20% for hospitalized patients and as high as 50% during epidemics [4,5]. Presently, there is no licensed vaccine or immunotherapy available for preventing or treating this disease. Although the antiviral drug Ribavirin is somewhat beneficial, it must be administered at an early stage of infection to successfully alter disease outcome, thereby limiting its utility [6]. Furthermore, there is no commercially available Lassa fever diagnostic assay, which hampers early detection and rapid implementation of existing treatment regimens (e.g. Ribavirin administration). The severity of the disease, ability to be transmitted by aerosol, and lack of a vaccine or therapeutic drug led to its classification as a National Institutes of Allergy and Infectious Diseases (NIAID) Category A pathogen and biosafety level-4 (BSL-4) agent.

The LASV genome is comprised of two ambisense, single-stranded RNA molecules designated small (S) and large (L) [7]. Two genes on the S segment encode the nucleoprotein (NP) and two envelope glycoproteins (GP1 and GP2); whereas, the L segment encodes the viral polymerase (L protein) and RING finger Z matrix protein. GP1 and GP2 subunits result from post-translational cleavage of a precursor glycoprotein (GPC) by the protease SKI-1/S1P [8]. GP1 serves a putative role in receptor binding, while the structure of GP2 is consistent with viral transmembrane fusion proteins [9]. NP is an abundant virion protein that binds and protects the viral RNA. The Z matrix protein associates with GP2 and NP during viral biogenesis, but alone is sufficient to mediate formation and release of viral particles from infected/transfected cells [10].

## Results

### LASV gene expression and incorporation in VLP

**Figure 1:**
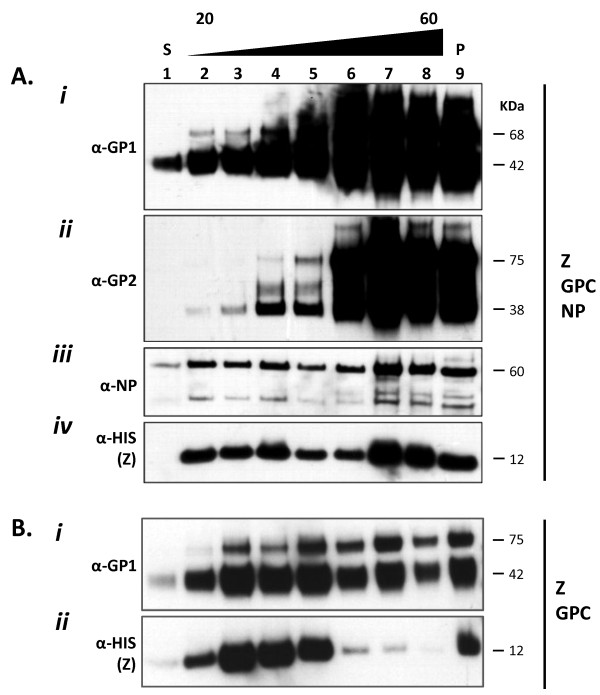
**Purification of HEK-293T/17 generated LASV VLP by sucrose gradient sedimentation and detection of GP1, GP2, NP, and Z proteins in fractions by western blot analysis**. LASV VLP were precipitated with PEG-6000/NaCl and concentrated by ultracentrifugation. Pellets were resuspended in 500 μL of TNE or PBS, overlayed on discontinuous 20 - 60% sucrose gradients, and sedimented by ultracentrifugation. Eight fractions of 500 μL each were collected from sucrose gradients. Ten μL from each fraction were separated on denaturing 10% NuPAGE gels, blotted and probed with LASV protein-specific mAbs. LASV VLP packaging Z+GPC+NP (A) and Z+GPC (B) were analyzed for distribution of GP1 (A*i*, B*i*), GP2 (A*ii*), NP (A*iii*), and Z (A*iv*, B*ii*) throughout the gradient spectrum. Fraction 1 contained input supernatant (S) loaded onto gradients. Fractions 2 through 8 were from 20 - 60% sucrose gradients. Lane 9 contained insoluble material that pelleted through 60% sucrose (P). The size of each protein in kDa is indicated to the right of each blot (unprocessed GPC: 75 kDa, GP1: 42 kDa, GP2: 38 kDa, NP: 60 kDa, and Z: 12 kDa).

Transient transfection of HEK-293T/17 cells with LASV GPC, NP, and Z gene constructs resulted in high level expression of all proteins, including their known post-translational processing. The glycoprotein complex (GPC) was detected as a 75 kDa polyprotein precursor in transfected cell extracts, and in VLP preparations (Figure 1 A*i*, A*ii*, B*i *lanes 2 - 9; Additional file 1: Figure S1 C*i *lane 4). Similarly, the proteolytically processed GP1 and GP2 subunits were detected in cell extracts (Additional file 1: Figure S1 C*i *lane 4) and in purified VLP (Figure 1 A*i*, A*ii*, B*i *lanes 2 - 9) as 42 and 38 kDa glycosylated species, respectively. In VLP cell culture supernatants cleared by ultracentrifugation, the soluble LASV GP1 isoform previously described in this expression system was also detected at high levels (Figure 1 A*i*, lane 1) [11,12]. Nucleoprotein (NP) was mainly detected as a 60 kDa species with smaller fragments identified, namely a 24 kDa protein corresponding to a previously described proteolysis product generated during LASV infection *in vitro *(Figure 1 A*iii *lanes 2 - 9; Additional file 1: Figure S1 C*i*, lane 1), [13-16]. The nucleoprotein was largely absent from the extracellular milieu (Additional file 1: Figure S1 C*ii*, lane 1) unless the Z matrix protein was co-expressed (Figure 1 A*iii*, A*iv*, lanes 2 - 9). Nucleoprotein that was not associated with VLP was present in the input fraction, as assessed by corresponding lack of GP2 and Z matrix protein detection (Figure 1 A*iii*, lane 1). The Z matrix protein was detected in cell extracts (Additional file 1: Figure S1 C*i*, lane 2) and in VLP preparations, as a 12 kDa protein (Figure 1 A*iv*, B*ii*, lanes 2 - 9). An N-terminal 6X-HIS tagged Z protein gene variant starting at amino acid position +3 that disrupted the known mirystoylation domain also expressed at high levels, but failed to generate VLPs, as determined by lack of detection of the protein in cell culture supernatants (Additional file 1: Figure S1 C*i*, *ii*, lane 3).

To determine if tagged arenaviral gene sequences benefitted overall expression levels and incorporation into VLP a series of matrix experiments were performed that combined native and/or 6X-HIS or FLAG epitope tags. Only the addition of a 6X-HIS tag to the C-terminus of the Z gene did not affect its expression and incorporation into VLP (Additional file 2: Figure S2). The addition of C-terminal tags to GPC or NP resulted in lower expression levels and resulting incorporation into VLP. In some cases these tags led to unexpected and untoward proteolytic processing (Additional file 2: Figure S2, lane 6).

### Large scale generation of LASV VLP

Generation of LASV VLP from 6 well plates through 15 cm cell culture dishes resulted in linear volumetric increase in particle yields (~100 μg/35 mm well; ~2 mg/15 cm dish). Production of VLP for biochemical characterization and *in vivo *studies was performed in multiple 15 cm culture dishes, which routinely yielded an average of 2 mg of total VLP protein per dish, as determined by Micro BCA and SDS-PAGE. VLP generated from expression of LASV Z, GPC, and NP gene constructs resulted in particles with higher densities than those produced by expression of Z and GPC alone, as assessed by relative levels of each viral protein throughout the sucrose density spectrum (Figure 1A,B, lanes 2 - 9). The majority of Z+GPC+NP VLP sedimented between 30 and 60% sucrose (Figure 1A*i *- *iv*, lanes 4 - 8), whereas Z+GPC VLP were present in ~25 - 40% sucrose fractions (Figure 1B*i*, *ii*, lanes 3 - 5). Surprisingly, Z+GPC VLP sedimenting through 30 - 60% sucrose contained progressively lower levels of Z matrix protein (Figure 1B*ii*, lanes 6 - 8) than counterparts containing both NP (Figure 1A*iv*, lanes 6 - 8) and Z. In both Z+GPC and Z+GPC+NP VLP preparations a considerable insoluble fraction pelleted through 60% sucrose, and could only be dissolved in reducing SDS-PAGE buffer (Figure 1A*i *- *iv*, 1B *i *- *ii*, lane 9 [P]).

### Effects of LASV gene expression on mammalian cell morphology - cytotoxicity

**Figure 2:**
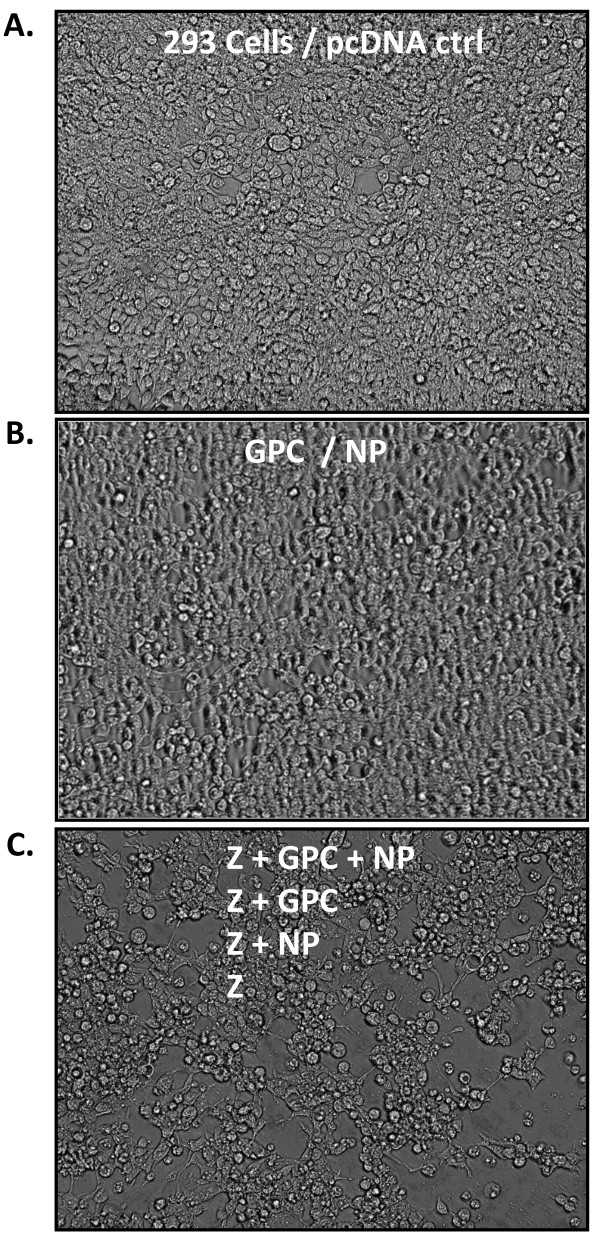
**Light microscopy analysis of HEK-293T/17 cells transfected with LASV gene constructs**. Representative fields of untransfected or vector control transfected (A), LASV NP or GPC (B), or Z, Z+GPC, Z+NP, Z+GPC+NP (C) transfected HEK-293T/17 cells at 72 hours photographed in 6-well plates at 400X magnification are shown. Control or single gene transfected cells retain fibroblastic shape in undisturbed monolayers (A and B). By contrast, any combination of LASV gene constructs that include the Z matrix protein result in loss of fibroblastic cell shape, with pronounced rounding and detachment from the Poly-D-Lysine coated plastic surface, resulting in significant disturbance in the monolayer (C).

Expression of LASV GPC or NP alone did not induce significant morphological changes in 293T/17 cells through 72 hours post-transfection when compared to untransfected, mock transfected, or vector only transfected cells, as assessed by light microscopy (Figure 2A,B). By contrast, inclusion of Z matrix gene protein in transfection experiments resulted in significant morphological changes marked by elongation of cells by 24 hours and significant detachment from the Poly-D-Lysine coated culture surface by 48 hours, resulting in large areas of monolayer breakdown (Figure 2C). Cellular cytotoxicity was measured by MTT assays, and chromosomal DNA fragmentation analysis was employed to determine gross apoptotic or necrotic cell death mechanisms. Triplicate MTT experiments verified that single LASV NP, GPC, and GPC-FLAG gene expression did not result in significant cellular cytotoxicity when compared to vector transfected and untransfected 293T/17 cell controls (Additional file 3: Figure S3B, lanes 1 - 3 versus lanes 16, 17). The inclusion of LASV Z or Z3'HIS in transfections experiments, alone or in combination with any other LASV gene constructs resulted in significant levels of cytotoxicity, as measured by reduced O.D. 562 levels in MTT assays (Additional file 3: Figure S3, lanes 4 - 15), with p < 0.05 to p < 0.001, n = 3 for each condition. Despite significant differences in MTT assays among transfected LASV gene combinations, TAE-agarose gel analysis showed lack of visible DNA fragmentation after a 72 hour transfection (Additional file 3: Figure S3A, lanes 4 - 17).

### LASV VLP contain a multitude of cellular proteins in addition to viral polypeptides

**Figure 3:**
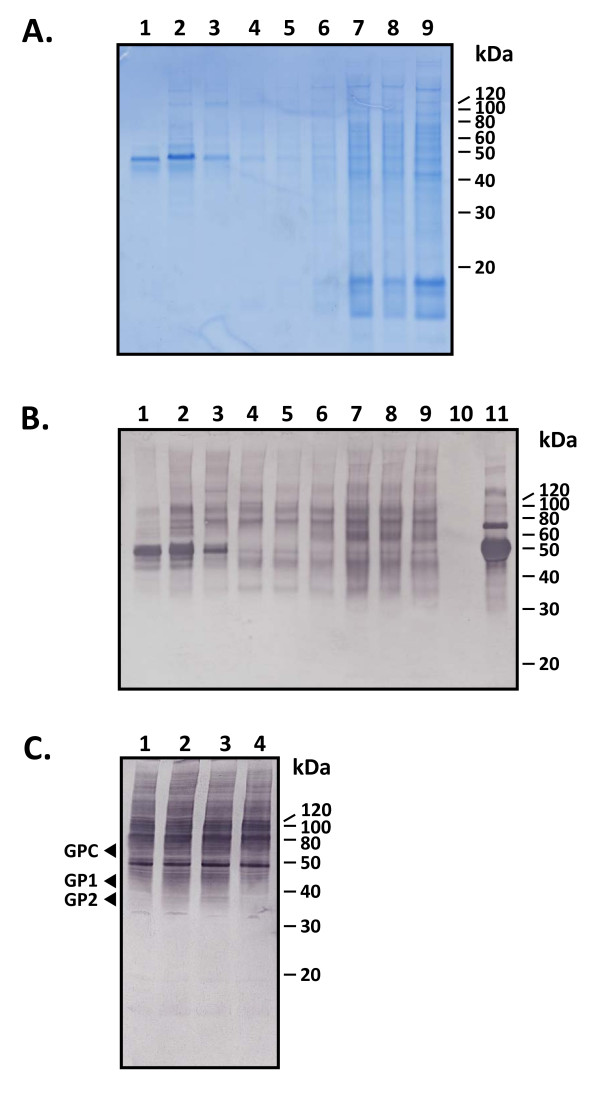
**Lectin binding profiles on sucrose purified VLP**. LASV Z+GPC+NP VLP fractions obtained from sucrose gradient sedimentation corresponding to those in Figure 1A were subjected to SDS-PAGE (3A) and lectin binding analysis on proteins transferred to nitrocellulose membranes (3B). A combination of agglutinins, GNA (*Galanthus nivalis*), SNA (*Sambucus nigra*), MAA (*Maackia amurensis*), PNA (*Peanut*), and DSA (*Datura stramonium*), were combined and used to probe VLP fractions 1 through 9 (3B, lanes 1 - 9). LASV NP, GP1, and GP2 generated in *E. coli *were used as unglycosylated protein controls (3B, lane 10). A combination of four glycoproteins was used as positive controls for lectin binding: carboxypeptidase Y (63 kDa), transferrin (80 kDa), fetuin (68, 65, 61 kDa), and asialofetuin (61, 55, 48 kDa) (3B, lane 11). For visual comparison purposes an SDS-PAGE gel was run with the same VLP fractions, stained with Coomassie BB-R250, and photographed (3A, lanes 1 - 9). LASV Z, Z+GPC+NP, Z+GPC, Z+NP VLP purified through 20% sucrose cushions were similarly analyzed for glycan binding (3C, lanes 1 - 4, respectively). The relative positions of GPC, GP1, and GP2 are noted to the left of the gel. Protein molecular weights in kDa are noted to the right of each image.

Analysis of sucrose gradient-purified LASV VLP by SDS-PAGE and Coomassie BB-R250 staining revealed a multitude of proteins in addition to the expected viral polypeptides at ~ 40 kDa (GP1 and GP2), 60 kDa (NP), and 12 kDa (Z) (Figure 3A, lanes 1 - 9). These additional proteins are host cell derived polypeptides which range from ~20 kDa to 200 kDa in size. Supernatants of mock or pcDNA3.1+:intA transfected cells do not yield detectable levels of PEG-6000/NaCl and sucrose cushion and/or gradient centrifugation-derived proteins, as determined by Micro BCA and SDS-PAGE analyses (data not shown). Glycan analysis using a wide range of lectins revealed that a significant number of non-viral proteins incorporated into LASV VLP are glycoproteins (Figure 3B, lanes 1 - 9). Lectin binding specificity was assessed by lack of binding to LASV NP, GP1, and GP2 proteins generated in *E. coli *(Figure 3B, lane 10). Lectin binding to glycosylated proteins included in the DIG Glycan Differentiation Kit was included as a positive control (Figure 3B, lane 11). A similar lectin binding analysis was obtained with VLP purified through 20% sucrose cushions containing Z alone, Z+GPC+NP, Z+GPC, or Z+NP (Figure 3C, lanes 1- 4, respectively), with the exception that additional diffuse bands could be discerned in VLP containing LASV glycoproteins (Figure 3C, lanes 2, 3).

### LASV VLP glycoproteins display heterogeneous glycosylation

**Figure 4:**
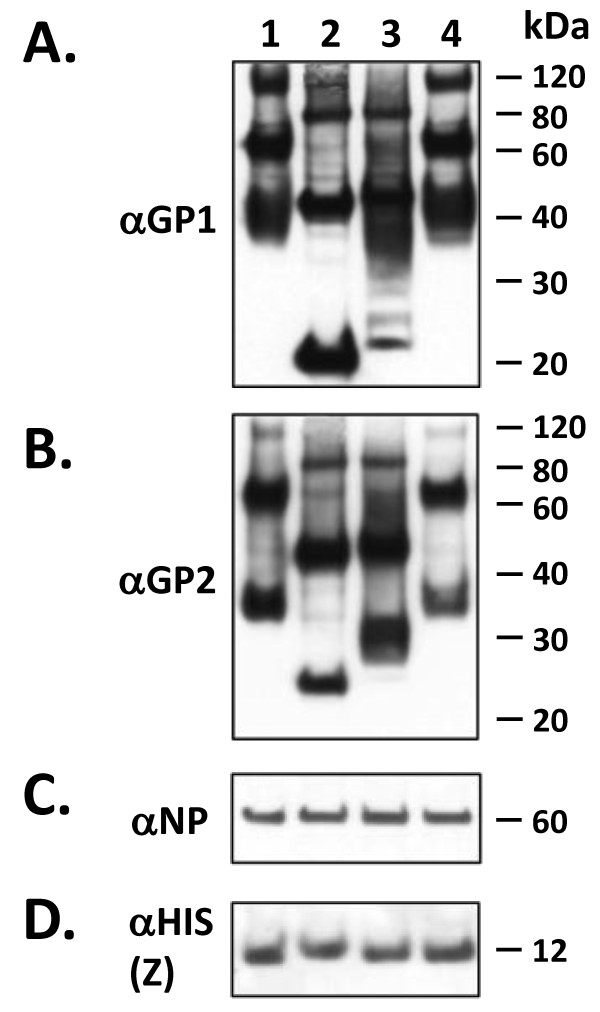
**Deglycosylation analysis of LASV Z+GPC+NP VLP**. Non-denatured LASV Z+GPC+NP VLP were subjected to deglycosylation with PNGase F (4A - D, lane 2), Endo H (4A - D, lane 3), neuraminidase (4A - D, lane 4), or were left untreated (4A - D, lane 1), followed by SDS-PAGE and western blot analyses. Blots were probed with α-GP1 (4A), α-GP2 (4B), α-6X-HIS (Z) (4D) mAbs, or α-NP PAb (4C). PNGase F completely deglycosylated both GP1 and GP2 (4A, 4B, lane 2, respectively), resulting in a mobility shift of both proteins corresponding to their unprocessed polypeptide backbone molecular weights, of 20 kDa and 23 kDa, respectively. Conversely, Endo H showed little affect of GP1 (4A, lane 3) but significantly deglycosylated GP2, generating a relatively uniform, partially glycosylated species of ~ 30 kDa (4B, lane 3). Following Endo H digestion, which cleaves high mannose and some hybrid oligosaccharides from the backbone of N-linked glycoproteins, ~ 7 kDa of the GP2 mass remains inaccessible to this enzyme. Similar results were obtained when pre-denatured VLP were used as input in the reaction. Neuraminidase had no affect on the glycosylation profile of GP1, GP2, or GPC (4A, 4B, lane 4). None of the deglycosidases affected the mobility of NP (4C, lanes 2 - 4) or Z (4D, lanes 2 - 4) proteins. Protein molecular weights in kDa are noted to the right of each blot.

LASV VLP containing Z+GPC+NP were treated with PNGase-F, Endo-H, or neuraminidase to assess gross glycosylation patterns. Experiments were performed with non-denatured (Figure 4) and with heat denatured VLP (data not shown), with identical results. PNGase-F completely removed glycans from GP1 and GP2, as well as from unprocessed GPC, as determined by mobility shifts from 42 to 20 kDa for GP1, 38 to 22 kDa for GP2, and from 75 to 42 kDa for GPC (Figure 4A,B, lane 2). By contrast, Endo-H removed glycans from GP1, but to a much lesser extent than from GP2. Multiple bands were detected with α-GP1 mAb in Endo-H treated LASV VLP containing GPC, ranging between 22 and 42 kDa, whereas probing of the same reactions with α-GP2 mAbs revealed a relatively homogeneous GP2 species at approximately 30 kDa (Figure 4A,B, lane 3). Treatment of LASV VLP with neuraminidase resulted in GP1 and GP2 glycosylation patterns similar to those obtained with untreated VLP (Figure 4A,B, lane 4 versus lane 1). Treatment of LASV VLP with all three deglycosydases did not affect the mobility of NP (Figure 4C, lanes 1 - 4) and Z proteins (Figure 4D, lanes 1 - 4). In addition to deglycosylation of monomeric glycoproteins and unprocessed GPC, mobility shifts were readily detected for the approximately 120 kDa species likely composed of previously characterized trimerized glycoproteins monomers resistant to denaturation with SDS, reducing agents, and heat (Figure 4A,B, lanes 3, 4) [11,12].

### LASV VLP do not package cellular ribosomes

**Figure 5:**
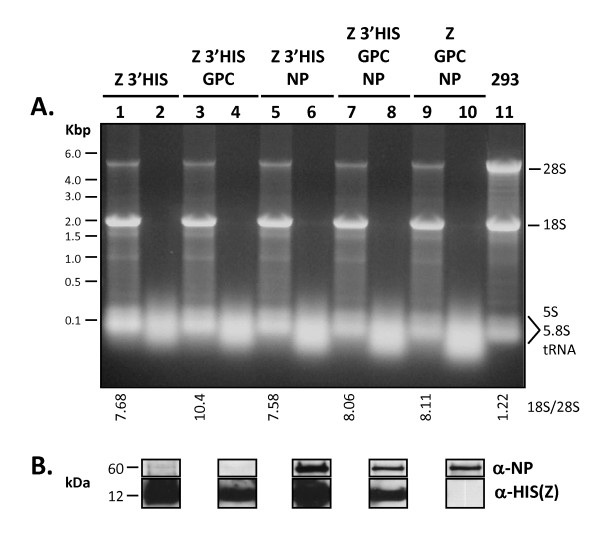
**Analysis of RNA content in LASV VLP and corresponding transfected HEK-293T/17 cells**. RNA was isolated from the total VLP fraction generated in a single 10 cm cell culture dish (~ 6 ×10^7 ^cells), and the entire nucleic acid pellet was resolved on denaturing glyoxal agarose gels. RNA from Z3'HIS, Z3'HIS+GPC, Z3'HIS+NP, Z3'HIS+GPC+NP, and Z+GPC+NP (lanes 2, 4, 6, 8, and 10, respectively [V]), and 5 μg of total RNA isolated from the corresponding transfected HEK-293T/17 cells (lanes 1, 3, 5, 7, and 9, respectively [C]) were resolved per lane of a 1.5% gel. Untransfected HEK-293T/17 cell RNA was run alongside test samples as a control (lane 11 [C]). All VLP samples were devoid of rRNAs (28S ~5.5 kbp; 18S ~ 1.8 kbp), but all contained low molecular weight RNA species corresponding in size to tRNAs, approximately 50 - 100 nucleotides in length (lanes 2, 4, 6, 8, 10). Transfected cells producing LASV VLP showed a significant reduction in the 28S rRNA species (lanes 1, 3, 5, 7, 9) when compared to untransfected control cells (lane 11). Ratios of 18S/28S RNA in transfected and untransfected cells, determined by densitometry, are shown below panel A. Molecular weight sizes ranging from 0.5 - 6 kbp are noted to the left of the gel. The positions of cellular 28S and 18S ribosomal RNAs, and tRNA are noted to the right of the gel.

Ribonucleic acid content in LASV VLP generated in HEK-293T/17 cells lacked 18S and 28S ribosomal RNA (rRNA) species, as assessed by denaturing agarose gel electrophoresis, irrespective of the LASV gene combination (Figure 5A, lanes 2, 4, 6, 8, 10). A low molecular weight RNA species, approximately 75 base pairs or less, corresponding in size range to cellular tRNAs could be readily detected in VLP preparations containing either Z alone, or in combination with NP and GPC (Figure 5A, lanes 2, 4, 6, 8, 10). This species was not detected in mock or pcDNA3.1+:intA transfected cell supernatants extracted with Trizol reagent (data not shown). The 28S and 18S ribosomal RNA bands were present in total cellular fractions obtained from cells transfected with varying LASV gene constructs, although 28S/18S ratios were significantly reduced when compared to the pcDNA3.1+:intA transfected cell control (Figure 5, lanes 1, 3, 5, 7, 9, versus lane 11). To verify that input LASV VLP used in RNA analysis contained the respective viral proteins, aliquots of purified pseudoparticles were subjected to western blots analysis with α-NP, α-HIS (Z), and α-GP2 antibodies. Western blot analysis revealed that input LASV VLP expressed the respective proteins of interest (Figure 5B, lanes 2, 4, 6, 8, 10).

### LASV VLP are morphologically similar to native virions

**Figure 6:**
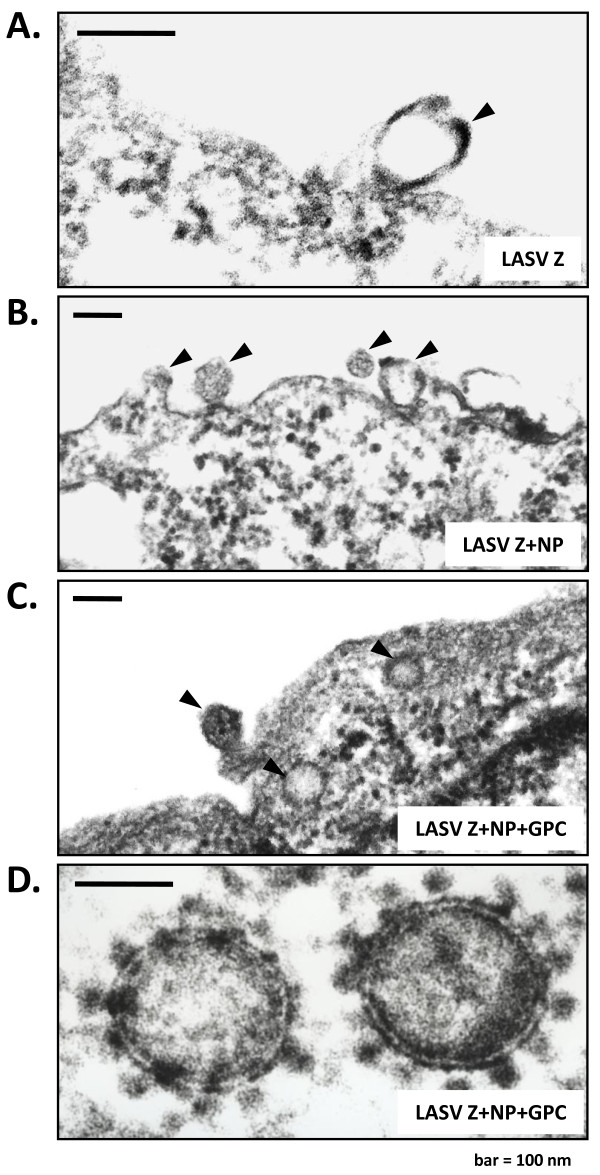
**Electron micrographs of LASV VLP budding from the surface of HEK-293T/17 cells expressing LASV Z alone or in combination with GPC and NP genes, and high magnification of LASV pseudoparticles**. Cells expressing LASV Z (6A), Z+NP (6B), or Z+NP+GPC (6C) were harvested at 72 hours post transfection, fixed in glutaraldehyde, and embedded in agarose plugs. Cell pellets were processed for EM analysis and were imaged. Images were printed on photographic paper and were subsequently scanned and saved as high resolution tiff files. LASV Z VLP budded from the surface of cells as empty particles, noted by the lack of electron dense cores (6A). By contrast, LASV Z+NP and Z+NP+GPC appear as electron dense particles containing subcellular structures (6A and 6B). LASV VLP budding from the surface of transfected cells or approaching the cell surface are marked by black arrows. Budded LASV Z+NP+GPC VLP appeared as round, dense structures enveloped in a bilayer structure, presumably a lipid envelope, and were associated with electron dense subcellular organelles (6D). These organelles were not identified as ribosomes in these studies. Cellular ribosomes are known to associate with and be packaged into native LASV virions. The bar in each Figure equals 100 nm.

Electron microscopy (EM) was employed to dissect the morphological properties of VLP generated by expression of Z matrix protein alone, or in combination with NP and GPC. Expression of LASV Z gene alone was sufficient to induce budding of low electron density empty VLP from the surface of transfected cells (Figure 6A). By contrast, expression of Z in conjunction with NP or NP+GPC resulted in the generation of electron dense VLP with granular material associated with the pseudoparticles (Figure 6B,C,D). The granular structures were similar in size to cellular ribosomes, or ~ 20 nm (Figure 6D), but identification of these subcellular organelles as the granular elements, as well as their physical association and incorporation in VLP were not investigated in these studies. LASV VLP displayed pleiomorphic morphology by EM, with sizes ranging from 100 - 250 nm, and enveloped by a bilayer structure (Figure 6D).

### LASV VLP display glycoprotein resistance to proteolysis by trypsin

**Figure 7:**
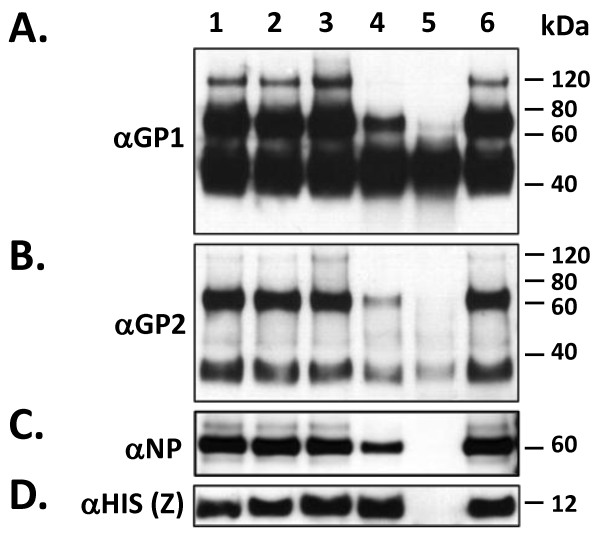
**Trypsin protection assay on LASV Z+GPC+NP VLP**. LASV VLP expressing Z, GPC, and NP proteins were subjected to trypsin protection assays to assess the enveloped nature of pseudoparticles and compartmentalization of viral proteins. LASV VLP incorporated unprocessed 75 kDa GPC precursor (7A, 7B, lane 1), and monomeric 42 kDa GP1 (7A, lane 1), and 38 kDa GP2 (7B, lane 1). LASV VLP also incorporated trimerized, non-reduceable 126 kDa GP1 isoforms (7A, lane 1), and 114 kDa GP2 trimers to a lesser extent (7B, lane 1). For trypsin protection assays ten μg of LASV VLP were either left untreated (lane 1), treated with 3 mg/mL soybean trypsin inhibitor (lane 2), 1% Triton X-100 (lane 3), 100 μg/mL trypsin (lane 4), 1% Triton X-100 and 100 μg/mL trypsin (lane 5), or 100 μg/mL trypsin in the presence of 3 mg/mL soybean trypsin inhibitor (lane 6). Trypsin alone completely digested trimerized GP1 (7A, lane 4) and GP2 (7B, lane 4), while partially degrading GPC precursor, but having little effect on monomeric glycoproteins. Trypsin treatment of intact VLP did not significantly affect the levels of NP (7C, lane 4), and Z (7D, lane 4) proteins. Treatment of VLP with Triton X-100 in the presence of trypsin resulted in the complete digestion of NP (7C, lane 5) and Z (7D, lane 5), while only partially degrading monomeric GP1 (7A, lane 5) and GP2 (7B, lane 5) proteins. Treatment of VLP with trypsin in the presence of soybean trypsin inhibitor completely prevented digestion of any form of all viral proteins (7A - 7D, lane 6).

Trypsin protection assays were employed to characterize protein content and structural compartmentalization of LASV antigens. Treatment of VLP with soybean trypsin inhibitor alone, with 1% Triton X-100 alone, or with soybean trypsin inhibitor and trypsin had no effect on the integrity of GP1, GP2, Z, and NP proteins when compared to untreated controls (Figure 7A - 7D, lanes 2, 3, 6 versus lane 1). Treatment of VLP with trypsin alone completely digested the approximately 120 kDa trimerized GP1 species and partially digested unprocessed GPC, while monomeric GP1 remained largely resistant to the protease (Figure 7A, lane 4). Similarly, trypsin completely digested the approximately 120 kDa trimerized GP2 species, but only partially digested monomeric GP2 (Figure 7B, lane 4). Trypsin treatment of intact LASV VLP did not significantly affect detection of NP and Z proteins (Figure 7C,D, lane 4). Whereas, treatment of LASV VLP with Triton X-100 and trypsin resulted in increased digestion of both glycoproteins, but significant levels of GP1 and GP2 could still be detected (Figure 7A,B, lane 5). Under these conditions, both NP and Z proteins were completely digested by trypsin (Figure 7C,D, lane 5). Digestion of intact VLP in the presence of soybean trypsin inhibitor completely prevented digestion of any form of the exposed glycoprotein complex (Figure 7A,B, lane 6).

### LASV VLP are immunogenic in mice and induce a mature IgG response after prime + two boosts intra-peritoneal immunizations

**Figure 8:**
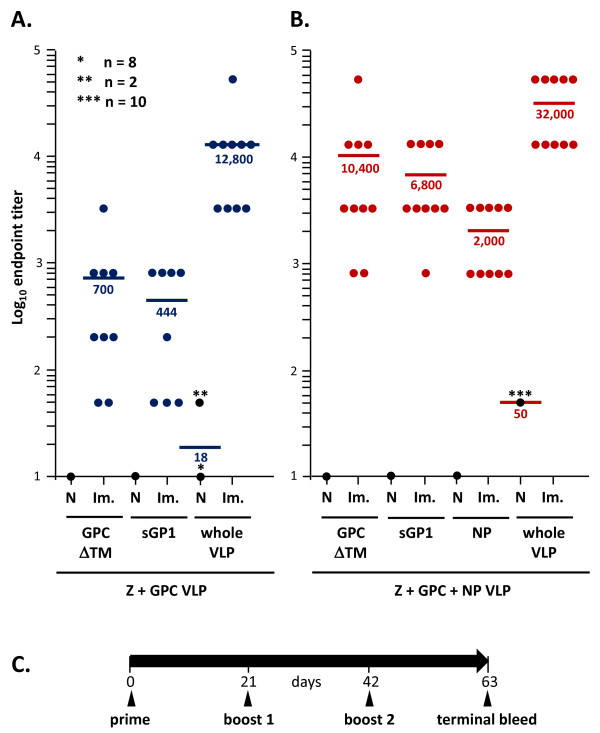
**Immunogenicity of LASV Z+GPC and Z+GPC+NP in a prime + 2 boosts regimen in BALB/c mice**. Groups of 10 BALB/c mice were immunized i.p. with either 100 μL of sterile TNE, or 10 μg of LASV VLP formulated in the same buffer using a prime + 2 boosts regimen, 3 weeks apart. Three weeks after the second boost all mice were sacrificed and sera were subjected to murine IgG endpoint titer determinations by ELISA on homologous VLP or recombinant LASV proteins coated on Nunc Maxisorp plates. Endpoint titers were calculated using background subtraction binding values generated with normal mouse sera on recombinant VLP and LASV proteins. LASV Z+GPC immunizations generated significant titers against whole VLP (mean = 12,800), but generally low titers to viral GP1 and GP2, with means of 444 and 700, respectively (8A). A similar immunization schedule with LASV Z+GPC+NP VLP resulted in significantly higher endpoint titers to both glycoproteins, with means of 6,800 and 10,400 for GP1 and GP2, respectively (8B), and to whole VLP (mean = 32,000). Significant IgG titers were also generated to NP (mean = 2,000). Endpoint titers generated by sham immunized murine sera to recombinant LASV proteins were at the lower limit of detection of the assay (mean = 10), with slight increased non-specific titers against Z+GPC VLP (mean = 18) and Z+GPC+NP (mean = 50). The immunization schedule used in these experiments is graphically outlined in 7C.

Mice were immunized with LASV VLP containing Z and the glycoprotein complex (Z+GPC), or including the NP protein (Z+GPC+NP), in the absence of an adjuvant, using a prime + 2 boosts schedule, 3 weeks apart. Total LASV antigen-specific IgG levels were assessed by ELISA on VLP, NP, GP1, or GP2 coated plates. Three weeks following a single 10 μg dose administration of VLP a significant number of mice had generated IgG-specific responses to LASV antigens (Table 1, pre-1^st ^boost column). Following a homologous first boost, all animals generated more robust LASV protein-specific IgG, which was further enhanced in all animals after a second boost, and assessed terminally 63 days post first immunization (Figure 8; Table 1). The IgG response against both types of whole VLP was significantly more robust than to individual antigens, with mean endpoint titers of 12,800 and 32,000 for Z+GPC and Z+GPC+NP VLP, respectively. Most notably terminal IgG titers against GP1 and GP2 in Z+GPC+NP VLP were approximately 15 fold higher than to Z+GPV VLP. Most animals immunized with Z+GPC VLP responded poorly to both glycoproteins, with 2/10 and 3/10 producing endpoint titers of 50 to GP2 and GP1, respectively, with only one animal registering an IgG titer of 3200 to GP2. Animals immunized with Z+GPC+NP responded well to both glycoproteins, with mean titers of 10,400 and 6,800 for GP2 and GP1, respectively, with 4/10 animals registering greater than 12,800 endpoint titer to each glycoprotein. Despite an increased response to GP2 in animals immunized with Z+GPC+NP statistical significance was not achieved versus the GP2 response to Z+GPC VLP (Table 1). Titers to Z matrix protein were not determined in these studies.

**Table 1:** Increasing IgG titers to Lassa virus antigens through the vaccination schedule

	Immunogen								
	Z+GPC VLP				Z+GPC+NP VLP				
ELISA Ag	naive	pre- 1^st^ boost	pre- 2^nd ^boost	term.	naive	pre- 1^st^ boost	pre- 2^nd ^boost	term.	p value
VLP	18 ± 17	556 ± 975	2667 ± 1058	12800 ± 14311	50 ± 0	9920 ± 4637	19520 ± 16963	32000 ± 20239	0.026
sGP1	< 10	88 ± 69	200 ± 254	444 ± 384 **	< 10	1520 ± 1159	2480 ± 1159	6800 ± 5215	0.004
GPCΔTM	< 10	95 ± 72	215 ± 217	700 ± 992 *	< 10	2960 ± 3657	3440 ± 3478	10400 ± 15179	0.092
NP					< 10	560 ± 310	1220 ± 1060	2000 ± 1265	

Endpoint ELISA titers for each timed point are mean ± SD, N = 10, except for starred entries, where * N = 9 and ** N = 8. The p value for terminal endpoint IgG titers generated against relevant LASV antigens between the two VLP formats is shown. Samples were collected on days 21 (pre-1^st ^boost), 42 (pre-2^nd ^boost) and 63 (term.) for titer analysis.

### LASV patient sera specifically recognize VLP antigens in conformational and individual recombinant viral proteins

**Figure 9:**
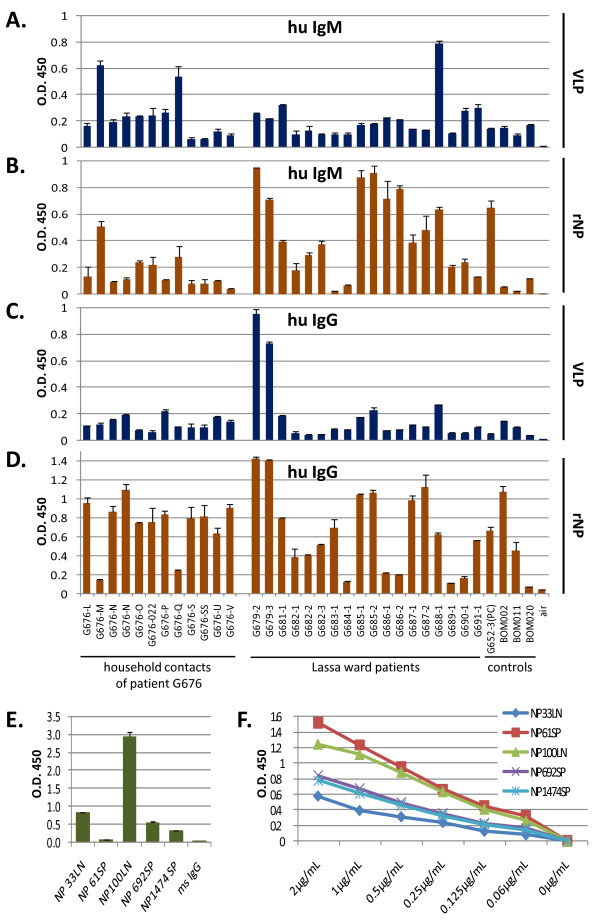
**Binding profile of human serum IgM and IgG, and NP-specific mAbs on LASV VLP and recombinant nucleoprotein**. Human sera collected from household contacts of patient G676, individuals hospitalized at the KGH at the time of analysis, or from supposedly LASV naive controls were diluted 1:100 in a proprietary sample diluent buffer containing 0.05% Tween 20 (Corgenix Medical Corp.) and assayed by ELISA on plates coated with 2 μg/mL total VLP protein (Figure 9A, 9C) or 2 μg/mL rNP (Figure 9B, 9D) per well. Detection of bound human IgM (Figure 9A, 9B) or IgG (Figure 9C, 9D) was performed as outlined in methods. LASV VLP captured IgM from three samples (G676-M, G676-Q, G688-1), all of which were also detected by rNP ELISA (Figure 9A, 9B), but did not result in binding by IgM from 14 additional samples that also tested positive on rNP (Figure 9A, 9B), including the G652-3 positive control. Similarly, VLP detected LASV-specific IgG in 2 samples (G679-2, G679-3), but did not identify 24 others detected in rNP ELISA (Figure 9C, 9D). For analysis of mAb binding profiles LASV VLP were coated in high protein binding ELISA plates at the same concentration as above. The indicated NP-specific mAbs were then used in a binding assay, at 1 μg/mL, alongside mouse IgG as a negative control (Figure 9E). For capture and detection of NP in solution, each NP-specific mAb was coated on ELISA plates at 5 μg/mL, followed by incubation with serial dilutions of nucleoprotein in sample diluent (Figure 9F). Captured NP was detected with a polyclonal Goat α-NP-HRP conjugate.

LASV-specific IgM and IgG titers in convalescent subjects and patient sera were used to characterize humoral responses to quasi-native viral epitopes on VLP. A subset of sera reacted with LASV VLP in either IgM or IgG detection platforms, but usually not both (Figure 9A,C). None of the presumed negative control samples showed reactivity to LASV VLP in these assays (Figure 9A,B, lanes BOM002, BOM011, BOM020). The positive control serum did not react with LASV VLP in the present format (Figure 9A,C, lane G652-3(PC)), although it bound to rNP in both IgM and IgG assays format (Figure 9B,D, lane G652-3(PC). Overall, there was poor correlation between LASV VLP and rNP detection of viral protein-specific IgG and IgM in human sera. Characterization of LASV NP epitope presentation in the context of a VLP was performed by ELISA using a series of mAbs raised against recombinantly expressed LASV NP. All five NP-specific mAbs showed differential binding levels to NP in VLP (Figure 9E), despite all capturing recombinantly expressed NP in solution at the concentration tested (Figure 9F).

## Discussion

Lassa virus-like particles were generated to contain the major immunological determinants of the virus, resembled native virions structurally, and were immunogenic in mice. Plasmid vectors well suited for high level expression of recombinant proteins in mammalian cells through combination of rational design and proven genetic elements have resulted in high yields of LASV VLP. These vectors afford the possibility of developing a VLP-based vaccine candidate in mammalian cell systems at low cost per dose, using transient expression technologies. Despite incorporation of all LASV proteins into VLP, both glycoproteins were present at significantly higher levels in most sucrose density fractions than either NP or Z (Figure 1). Incorporation of high levels of both glycoproteins in VLP may be beneficial in a vaccine platform, as these viral components alone have been shown to confer full protection against challenge with lethal doses of live LASV in non-human primates [17-21]. Yet, despite the high levels of glycoprotein incorporation into LASV VLP, addition of the nucleoprotein may be of critical importance in establishing more robust and long lived immunity against Lassa virus [19,22]. Previous studies have demonstrated physical interaction between the glycoprotein complex, the Z matrix, and nucleoproteins during viral biogenesis [23-25]. Thus, these natural interactions are greatly beneficial since they result in the generation of VLP that package all viral immunogenic and protective determinants from a single set of transiently transfected recombinant LASV genes. In these studies we employed the human endothelial kidney cell line HEK-293T/17 for its high levels of transfectability, expression of recombinant proteins from human cytomegalovirus (hCMV) promoter driven gene constructs, and resulting yields of LASV VLP. During the course of this work, we have also established the value of using HEK-293T/17 as an indicator cell line. The profound morphological changes manifested by the cell line upon expression of LASV Z matrix protein is a good indicator of transfection efficiency and overall production levels of resulting VLP (Figure 2). Despite significant adverse metabolic effects on cells expressing LASV proteins and generating budding VLP, culture viability remained high (mean = 70%) at the time of harvest. This desirable aspect of mammalian cell culture-based production is beneficial in downstream purification processes, by reducing host cell components that must be eliminated from the final purified product, namely the cellular proteins, DNA, RNA, and lipids. Other expression platforms cannot be easily employed in the generation of LASV VLP where the glycoprotein complex precursor is used to incorporate processed GP1 and GP2. Truncated versions of the GPC precursor lacking the transmembrane domain have been generated in *E. coli *(unpublished data from the Viral Hemorrhagic Fever Research Consortium) and in baculovirus expression systems [26]. In *E. coli*, the protein is neither glycosylated nor cleaved into GP1 and GP2 subunits. In insect cells, the protein is glycosylated but is not cleaved [26]. Both expression systems lack the critical SKI-1/S1P subtilase responsible for co-translational processing of the LASV GPC precursor in mammalian cells [27]. Despite the possibility of co-expressing the subtilase in heterologous systems to facilitate processing of GPC precursor, the glycosylation profile of GP1 and GP2 subunits may play a critical role in the structure and function of each protein *in vivo*. Thus, a mammalian expression system remains a highly attractive platform for the development of an arenaviral VLP-based vaccine.

We have determined in these studies that LASV VLP contain, in addition to the intended viral polypeptides, a plethora of host cell membrane proteins, presumably acquired during budding from the cell membrane or other intracellular lipid bilayer containing structures, such as the Golgi apparatus. A significant portion of the viral envelope protein content is made up of host cell glycoproteins, as determined by a broad glycan binding analysis performed on sucrose sedimented fractions (Figure 3B,C). The host cell glycoprotein composition varies along the gradient spectrum (Figure 3B). A similar pattern of cellular glycoproteins incorporated into LASV VLP was detected in purified particles generated from expression of Z alone or in combination with GPC and NP (Figure 3C). In Z+GPC or Z+GPC+NP VLP, a diffuse lectin binding pattern could be detected between 38 and 42 kDa which was absent from VLP that did not express the glycoprotein complex. This pattern was detected in addition to a prominent ~ 48 kDa cellular glycoprotein of unknown identity present in all VLP formats (Figure 3 C). The majority of detected cellular glycoproteins incorporated into LASV VLP ranged from 30 to greater than 220 kDa in mass. Recently, Moerdyk-Schauwecker et al. 2009 [28] characterized the spectrum of mammalian host cell proteins incorporated into vesicular stomatitis virus (VSV), an enveloped virus, during viral biogenesis. In total, 64 proteins of host cell origin were identified via a proteomics approach coupled with mass spectrometry (MS). Of the 64 host cell proteins identified in these studies, 10 were glycoproteins [28]. Although a similar study has not been performed for any member of the arenaviridae, it is likely that some common host cell proteins are packaged among a wide array of viral classes, and some of these proteins may even play functional roles during viral infection and replication. Characterization of the host cell protein profile in LASV VLP will be paramount in gaining regulatory clearance of an arenaviral pseudoparticle-based vaccine. The immunological and functional role of such proteins must be known in order to avert untoward side effects, such as autoimmunity and physiological disregulations.

We had previously characterized the gross glycosylation profile of LASV GP1 in the context of a soluble isoform (sGP1) of this viral protein [12]. In the present studies, we characterized LASV VLP-associated GP1 and GP2 glycosylation patterns. Glycoprotein 1 associated with VLP generated essentially the same glycosylation pattern as sGP1, with only partial deglycosylation by Endo H, and insignificant processing by neuraminidase (Figure 4A). These results point to a heterogeneous array of glycans on the surface of GP1 that include some high mannose and branched oligosaccharides. Glycoprotein 2 displayed a more heterogeneous glycan array with a highly homogeneous high mannose and hybrid oligosaccharide content that accounted for approximately 8 kDa of the fully processed mass of the protein, based on the detection of a relatively sharp 30 kDa species upon treatment with Endo H (Figure 4B, lane 3). The remaining 7 kDa of glycan content could be removed by treatment of the protein with PNGase F, but not with neuraminidase (Figure 4B, lanes 2 and 4). A similar micro- and macroheterogeneity in both GP1 and GP2 N-linked glycosylation has not been characterized in native Lassa virions.

Through these studies, we have established that GP1 incorporated into LASV VLP is highly resistant to proteolytic digestion by trypsin (Figure 7A, lanes 4 and 5), despite 13 predicted trypsin recognition sites on the polypeptide backbone (ExPASy proteomics server tools, PeptideCutter [29]). Similarly, GP2 is resistant to digestion with trypsin, albeit to a lesser extent than GP1, even after solubilization of the pseudoparticle envelope with Triton X-100 (Figure 7B, lanes 4 and 5). The PeptideCutter tool in ExPASy predicted 25 recognition sites with high confidence in the GP2 polypeptide backbone. However, since the glycoprotein complex spike is the viral antigen most readily accessible to the innate immune system and to circulating serum proteases, it is likely that this molecule evolutionarily developed a significant level of proteolytic resistance in the structure-function relationship. It is of paramount importance to the virus that the critical components required for binding and fusion to permissive host cells be preserved. The specific glycosylation patterns on GP1 and GP2 may play a functional role in the observed resistance to proteolytic degradation. In the studies by Schlie et al. 2010 [23], Proteinase K protection assays performed on GP VLP also revealed partial resistance of the GP2 component against degradation by the protease, although solubilization with Triton X-100 in conjunction with protease resulted in complete digestion of the protein. Glycosylation of arenaviral glycoproteins is critical for protein stability, as unglycosylated GP1 and GP2 generated in *E. coli *are insoluble and require detergents, zwitterions, and reducing agents to remain in solution [11,30], and deglycosylating mammalian cell generated GP1 generally produces similar results (unpublished data).

To characterize the structural compartmentalization of viral proteins in LASV we performed trypsin protection assays in the absence or presence of the anionic detergent Triton X-100 (Figure 7). In the absence of detergent, trypsin completely digested non-reduceable GP1 trimer, partially degraded unprocessed GPC, but had no effect of monomeric GP1 (Figure 7A, lane 4). A similar digestion pattern was obtained for GP2 (Figure 7B, lane 4). The addition of detergent to the reaction enhanced digestion of unprocessed GPC and had a minor effect on sensitivity of GP1 to the protease (Figure 7A, lane 5). Dissolution of the envelope by detergent resulted in more pronounced degradation of GP2 by trypsin, although a significant portion of the monomer could be detected (Figure 7B, lane 5). Only treatment of LASV VLP with Triton X-100 resulted in proteolytic degradation of both Z matrix and NP proteins. These results strongly support the model of a LASV VLP containing glycoprotein spikes on the surface of a lipid envelope with an internal matrix of Z protein containing the nucleoprotein component. We have shown that the viral proteins NP, Z, GP1 and GP2 can be co-expressed in VLP. Protein-protein associations appear to be an important aspect to the formation of VLP. Schlie et al. 2010 [23] reported that a co-localization of NP, Z, and GP occurs near the nucleus. Similarly, Eichler et at. 2004 [24] demonstrated that NP and Z co-localize in the cell. They also demonstrated that NP could be precipitated using an antiserum against Z and vice versa. Furthermore, Schlie et al. 2010 [23] determined that NP did not influence the interaction of GP and Z, nor could an interaction between NP and GP be detected in the absence of Z in co-localization and immunoprecipitation experiments. However, pull down experiments performed by Schlie et al. 2010 [23] demonstrated an association between Z and GP and Z and NP. Strecker et al. 2006 [25] reported that Z myristoylation is important for binding to lipid membranes. Flotation experiments using wild-type Z protein and a mutant of Z at the myristoylation site showed that the mutant remains localized in the cytosol, whereas the wild-type associated with the membrane. Thus, the interactions between Z and the membrane and with GP and NP result in VLP formation with relevant proteins incorporated in virions.

Another structural component of native LASV virions are host cell ribosomes that are packaged during virion assembly, presumably for enhanced viral mRNA translation in the early stages of cellular infection. To determine whether LASV VLP containing any combination of Z matrix, GPC, and NP proteins mediated the ability to package cellular ribosomes, total RNA was isolated from pseudoparticles and analyzed by denaturing RNA gel electrophoresis (Figure 5). RNA was also isolated from the corresponding transfected cells and analyzed alongside VLP RNA. All VLP formats analyzed in these studies did not contain significant levels of the 28S and 18S ribosomal RNA species known to be critical components of mammalian ribosomes (Figure 5, lanes 2, 4, 6, 8, 10). In some analyses, RNA was purified from 1 mg of total purified VLP, and the entire purified nucleic acid fraction was analyzed by gel electrophoresis without distinct ribosomal RNA bands visible (data not shown). Despite the lack of rRNA detection in LASV VLP, all pseudoparticle formats analyzed in these studies contained significant levels of low molecular weight RNA species ~ 75 - 200 nt, that co-migrated with cellular 5S (120 nt) and 5.8S (160 nt) rRNA, and transfer RNAs (75 - 95 nt). It is reasonable to assume that in native VLP the incorporation of host cell ribosomes would result in the co-packaging of critical tRNAs for translation of viral mRNAs. Although in these studies the exact nature of the packaged RNA species was not characterized in detail, the results suggest that multiple RNA species of ribosomal origin are incorporated into VLP. To confirm that ribonucleoproteins were not incorporated into virions, we performed western blot analysis on VLP proteins using antibodies raised against U1 snRNP 70, La/SSB, and Ro/SSA. No ribonucleoproteins could be detected in pseudoparticles (data not shown). These studies also point to a critical presence of viral RNA polymerase and genomic RNA segments during replication for subsequent incorporation of host cell ribosomes into nascent viral particles. The lack of detectable ribosomes in LASV VLP represents a regulatory advantage for this platform as a vaccine candidate. Administration of pseudoparticles containing autologous ribosomes to vaccinees has potential to result in untoward immunological affects.

Despite the lack of detectable 28S and 18S rRNA in LASV VLP comprised of any combination of LASV proteins analyzed in these studies, pseudoparticles that contained GPC and/or NP in addition to Z matrix protein were morphologically similar to native virions (Figure 6B,C,D). These VLP were electron dense particles with punctuate inclusions and appeared to associate with highly electron dense subcellular organelles in the cytoplasm, possibly ribosomes despite their lack of incorporation into the pseudoparticle (Figure 6C,D). The size of mammalian ribosomes is approximately 20 nm, in line with the size of the particles associated with nascent LASV VLP imaged in these studies (Figure 6D). However, these subcellular structures could not be detected in VLP budding from the surface of cells transfected with Z matrix protein alone (Figure 6A), which appeared empty and containing only an envelope structure, as shown here and reported by others [31].

For immunizations, LASV VLP comprised of Z+GPC or Z+GPC+NP were formulated in PBS and used to immunize BALB/c mice, in a prime + 2 boosts schedule, 3 weeks apart, in the absence of an adjuvant, and administered by i.p. injection. After a single immunization some animals showed a low level IgG response to individual LASV antigens, with increasing mean antibody titers after each subsequent boost (Table 1). ELISA analysis of terminal IgG titers showed a clear difference in the response levels against GP1, and whole VLP between Z+GPC and Z+GPC+NP pseudoparticles (p = 0.004 and 0.026, respectively) (Figure 8A,B). VLP containing all three proteins induced a significantly higher response to the glycoprotein components compared to Z+GPC VLP, with a 15 fold overall increase in titer against both GP1 and GP2, despite a not quite significant statistical difference in the GP2 titers (p = 0.092). Likewise, the titers against whole Z+GPC+NP VLP were nearly 3 fold higher than to Z+GPC pseudoparticles (Figure 8A,B).

Lastly, we attempted to use LASV VLP as a diagnostic tool for the detection of viral protein-specific IgM and IgG in the serum of convalescent subjects, patients from the Lassa ward, contacts from patients who succumbed to Lassa fever, and individuals not known to have had the febrile illness at any given time in their lives. The LASV antigen binding profile of these sera was extensively characterized using highly sensitive and specific recombinant protein-based diagnostics under development by the Viral Hemorrhagic Fever Research Consortium. The overall poor level of correlation observed in human serum IgM (r = 0.3297; r^2 ^= 0.1087) and IgG (r = 0.6284; r^2 ^= 0.3949) binding profiles between LASV VLP and recombinant proteins in these studies was not surprising. Recombinant LASV proteins currently employed in diagnostic assays are generated in bacterial or mammalian cell systems, as outlined in Branco et al., 2008 [12], and Illick et al., 2008 [11]. Individually produced, purified, and characterized proteins are used alone or in combination to coat high protein binding ELISA plates for determination of serum IgM and IgG binding profiles. Thus, it would be expected that protein-protein interactions known to play a role during viral biogenesis and in the formation of LASV VLP result in presentation of different epitopes and conformations than in counterparts generated as individual polypeptides. The known interactions between Z, GPC, and NP proteins in a VLP format likely mask the presentation of relevant epitopes to which a given individual may have generated IgM and IgG. As a result, native presentation of antigens in the context of a VLP, even in the presence of low levels of the membrane solubilizing detergent Tween 20, will likely not result in disruption of protein interactions necessary for the detection of epitope-specific serum antibodies. This is supported by the fact that all five NP-specific mAbs used in this analysis detected and captured recombinantly expressed NP in solution (Figure 9F), albeit at different levels. In combination, these results strongly suggest that LASV proteins in the context of a VLP display epitopes that possibly mimic native conformation and presentation. These observations further support the use of LASV VLP as a vaccine platform by supplying a quasi-native antigen, thus allowing the innate and adaptive immune systems to preferentially target epitopes relevant for immune protection against the virus. In addition, the use of pseudoparticles in clinical assays may offer advantages over the use of recombinantly expressed individual proteins. Immune responses to LASV VLP may be directed against epitopes that are best or exclusively displayed in the context of a quasi native particle containing proteins assembled in a manner similar to functional viral biogenesis.

VLP have gained significant momentum in the past decade as premier vaccine platforms. The approval of Merck & Co., Inc.'s Gardasil^(r) ^(Human Papillomavirus Quadrivalent [Types 6, 11, 16, and 18] Vaccine, Recombinant) by regulatory agencies heralded a new era in vaccinology, demonstrating that VLP are immunogenic, safe, and well tolerated in humans, and confer nearly complete protective immunity against homologous viral strains in canine models [32-38]. ENGERIX-B [Hepatitis B Vaccine (Recombinant)] is a recombinant VLP-like hepatitis B vaccine developed and manufactured by GlaxoSmithKline plc. These "Dane" particles, generated in yeast strains, are comprised of HbsAg and yeast phospholipids, and are subsequently harvested by gradient centrifugation and properly disulfide-linked *in vitro *[39]. These particles are highly immunogenic, safe, well tolerated, and very efficacious.

VLP-based vaccine candidates have also been developed and tested for their efficacy in preventing a wide array of viral conditions, such as Influenza [40-44], Ebola [45,46], Marburg [45,47], West Nile virus [48], Dengue [49], Respiratory Syncytial Virus (RSV) [50], HIV [51-56], and Hepatitis C virus [57-59], and the most recently reported case of Chikungunya [60]. VLP platforms currently being evaluated toward clinical licensure include Novavax's trivalent seasonal influenza vaccine. In recent Phase II clinical trials the vaccine was well tolerated and safe in adults age 60 and older and in healthy volunteers 18 to 48 years of age [61,62]. Thus, it is reasonable to employ similar strategies to develop a vaccine platform based on VLP that contain all the relevant immunological determinants that are known to confer protective immunity against this viral hemorrhagic fever. Studies are currently ongoing to determine the in vivo efficacy of LASV VLP in relevant *in vivo *models.

## Conclusions

The generation and characterization of a LASV VLP platform displaying all major immunological and protective determinants of the virus, with quasi-native morphological and protein association properties, that induced significant IgG titers in mice potentiate further development as a viable human vaccine platform.

Presently, there is no licensed vaccine or anti-viral therapy available for the prevention or treatment of this disease, and there is no commercially available Lassa fever diagnostic assay. The threat posed by LASV is heightened further by the potential use of the virus as a biological weapon, which is substantiated by the stability of the virion, demonstrated person-to-person transmission, the severity of disease, lack of therapeutic and prophylactic reagents, and the capacity for aerosolization. Collectively, these factors underscore the need for effective diagnostics, vaccines, and therapies against Lassa fever. The work performed in these studies is a first step toward resolving a public health crisis in Africa and bioterrorism concerns elsewhere.

## Methods

### Cells, plasmids, antibodies

HEK-293T/17 cells (ATCC CRL11268) were maintained in complete high glucose Dulbecco's Modified Eagle Medium (cDMEM) supplemented with non-essential amino acids (NEAA) and 10% heat-inactivated fetal bovine serum (ΔFBS).

Plasmid constructs expressing LASV GPC and the backbone vector pcDNA3.1+zeo:*int*A were described elsewhere [11]. Optimized Z and NP genes for expression were amplified from LASV Josiah infected VERO cell RNA, as previously outlined [11]. For immunoassays, Dr. Randal J. Schoepp kindly provided the LASV-specific GP1 mAb L52-74-7A and GP2 mAb L52-216-7, which were generated against purified gamma-irradiated LASV, as previously described [13]. Monoclonal antibody to poly-histidine (6X-HIS) was purchased from Invitrogen, Inc. LASV NP-specific polyclonal sera were generated in goats by immunizing animals with 100 μg of *E.coli *generated protein per injection, using a prime + 3 boosts strategy, followed by terminal bleeds (Bethyl Laboratories, Inc.). The LASV NP-specific goat IgG fraction was subsequently purified by affinity column chromatography with agarose beads coupled to NP immobilized by AminoLink chemistry (Thermo Fisher Scientific, Inc., Rockford, IL). Horseradish peroxidase (HRP)-conjugated secondary antibodies specific for goat and mouse IgG-gamma were purchased from Kirkegaard and Perry Laboratories (KPL, Gaithersburg, MD). The NP-specific hybridomas NP 33LN, NP 100LN, NP 61SP, NP 692SP, and NP 1474SP were generated by fusion of the SP2/0-Ag14 myeloma cell line with splenocytes and mesenteric lymph node lymphocytes from BALB/c mice immunized with *E. coli*-expressed NP, essentially as outlined by Köhler and Milstein [63-65]. Monoclonal antibodies were produced in serum free medium (PFHM II, Invitrogen), purified via Protein-G chromatography, quantitated by A280, BCA, and SDS-PAGE.

### Transient expression of LASV gene constructs

Recombinant LASV protein expression was analyzed in HEK-293T/17 cells transiently transfected with mammalian expression vector DNAs, which were prepared using the Endo-Free PureLink HiPure plasmid filter maxiprep kit (Invitrogen, Carlsbad, CA). Transfections and preparation of cell extracts for protein analysis have been described elsewhere [11]. The negative control vector pcDNA3.1(+):intA was included in all transfections. Protein concentration was determined for each sample by A280 with A260 subtraction, and verified using a Micro BCA(tm) Protein Assay Kit, as outlined by the manufacturer (Thermo Scientific).

### Generation and purification of LASV VLP

LASV VLP were generated by transfecting HEK-293T/17 cells in 6-well plates (for small scale analysis) or in 15 cm plates (for purification of multi-milligram quantities of VLP) using Lipofectamine 2000 (Invitrogen). Cells were seeded on plates coated with 50 μg/mL Poly-D-Lysine hydrobromide, and were transfected only at ≥90% confluence. Monolayers were transfected with equimolar amounts of vector DNAs, and when required reactions were normalized for DNA content with empty pcDNA3.1(+):intA. Cell supernatants were harvested 4 days post transfection and were clarified by centrifugation at 4000 ×g for 20 minutes at room temperature. Clarified supernatants were transferred to Beckman polyallomer ultratubes and gently mixed with polyethylene glycol-6000 (Sigma/Fluka) and sodium chloride to final concentrations of 5% and 0.25 M, respectively. Reactions were incubated at +4°C overnight, followed by centrifugation for one hour at 15,000 ×g, +4°C, in an SW28 rotor, to pellet the precipitated VLP. Pellets were gently resuspended in 20 mM Tris, pH7.4, 0.1 M NaCl, 0.1 mM EDTA (TNE), or in 1X PBS, pH 7.4, overlayed on 20% sucrose cushions, and centrifuged for 2 hours at 55,000 rpm, +4°C, in an SW60Ti rotor. Pellets were resuspended in TNE or PBS and VLP were further purified on 20 - 60% discontinuous sucrose gradients, as described above for sucrose cushions. VLP were removed from visible bands throughout the gradient, combined, diluted in TNE or PBS, and centrifuged for one hour at 15,000 ×g, +4°C, in an SW28 rotor, to pellet the purified VLP and to remove sucrose. Pellets were resuspended in TNE or PBS and allowed to dissolve fully at 4°C overnight. VLP used for immunizations were filtered through 0.45 μm syringe filters before being assayed for protein content by Micro BCA. VLP preparations were stored at 4°C in TNE or PBS at concentrations ranging from 200 - 3000 μg/mL. VLP for immunizations were tested for endotoxin levels with a high sensitivity *Limulus Amebocyte Lysate *(LAL) test (Sigma-Aldrich).

### Western blot and densitometry analyses

Expression of LASV GP1, GP2, NP, and Z-3'HIS in VLP were confirmed by Western blot analysis using anti-LASV mAbs L52-74-7A, L52-216-7, goat polyclonal antibody (PAb) to *E. coli *generated nucleoprotein and α-6X-HIS mAb, respectively. Secondary antibodies were horseradish peroxidase (HRP)-conjugated goat anti-mouse IgG (H+L) or rabbit anti goat IgG (H+L). Five to ten μg of total VLP protein were denatured, reduced, and resolved on 10% NuPAGE Novex Bis-Tris gels, according to the manufacturer's specifications (Novex, San Diego, CA). Proteins were transferred to 0.45-μm nitrocellulose membranes, blocked, and probed in 1X PBS, pH 7.4, 5% non-fat dry milk, 1% heat inactivated fetal bovine serum, 0.05% Tween-20, and 0.1% thymerosal. Membranes were then incubated in LumiGlo chemiluminescent substrate (KPL) and exposed to Kodak BioMax MS Film. Developed films were subjected to high resolution scanning for densitometry analysis. Quantification of band intensity was performed using National Institutes of Health ImageJ 1.41o software http://rsb.info.nih.gov/ij, and following the procedure outlined in http://www.lukemiller.org/journal/2007/08/quantifying-western-blots-without.html, using TIFF files.

### Cell proliferation assays

HEK-293T/17 cell cytotoxicity induced by LASV Z, GPC, and NP expression was monitored with a TACS(tm) MTT Cell Proliferation Assay (R&D Systems, Minneapolis, MN), according to manufacturer's instructions. The transfection procedure was scaled down to a 96-well format, with each condition analyzed in triplicate. Data was plotted as mean absorbance at 562 nm, with standard deviation, and background correction at 650 nm.

### Protease protection assays

Pseudovirus-specific protein composition and VLP structure were characterized by trypsin protection assays. Ten μg of purified VLP were treated with 100 μg/mL trypsin in the presence or absence of 1% Triton X-100, for 30 minutes, at room temperature. Reactions were stopped by the addition of soybean trypsin inhibitor to a final concentration of 3 mg/mL, addition of SDS-PAGE buffer and reducing agent (DTT), and heating to 70°C for ten minutes. Proteins were resolved on 10% NuPage gels and detected by western blot, as described above.

### PNGase F, Endo H, and neuraminidase assays

The glycosylation patterns of LASV VLP GP1 and GP2 generated from expression of LASV Z+GPC+NP were resolved by treatment with the deglycosidases PNGase F, Endo H, and neuraminidase, as previously described [12], on sucrose cushion purified VLP. Reactions were performed on heat denatured VLP to conform to manufacturer's recommendations for PNGase F and Endo H digestion conditions, and on non-denatured VLP. Control reactions were similarly processed except that enzymes were not added. Specificity of deglycosidases was assessed by monitoring the effects of all three enzymes on LASV NP and Z proteins packaged into VLP. Proteins were subsequently resolved by reducing SDS-PAGE, blotted, probed with α-LASV GP1, GP2, α-6X-HIS mAbs, or goat PAb α-NP, and developed as described above.

### Lectin-based Glycan differentiation assays

Glycosylation patterns of VLP associated proteins were characterized via binding of glycan-specific lectins using a DIG Glycan Differentiation Kit (Roche Applied Science, Mannheim, Germany), according to the manufacturer's instructions. LASV VLP proteins were resolved by reducing SDS-PAGE, blotted onto nitrocellulose, and subjected to lectin binding assays.

### RNA extraction from purified VLP

RNA was extracted from VLP with Trizol(tm) reagent/chloroform and isopropanol precipitation, essentially as outlined in the product insert (Invitrogen). RNA pellets were washed with 75% ethanol, air dried, resuspended in DEPC-treated water, and quantitated by A280. RNA was glyoxal-denatured and analyzed on 1.5% agarose gels containing ethidium bromide, essentially as described in Sambrook et al. [66]. Gels were photographed on a Kodak EDAS 120 system and images were saved as TIFF files for densitometry analysis. Total RNA was extracted from corresponding transfected HEK293T/17 cells using the same procedure.

### Genomic DNA fragmentation analysis

Genomic DNA was isolated from HEK-293T/17 cells using a Qiagen DNeasy kit, according to the manufacturer's instructions. Purified DNAs were quantitated by A260/A280. Two μgs of each DNA sample were resolved per lane of a 1.8% TAE/agarose gel containing 1 μg/mL ethidium bromide. High resolution gel images were converted to TIFF format for analysis.

### Murine immunizations

Six to eight week-old female BALB/c mice were purchased from Charles River Laboratories and housed according to Tulane University's IACUC guidelines. Research was conducted in compliance with the Animal Welfare Act and other Federal statutes and regulations relating to animals and experiments involving animals and adheres to principles stated in the Guide for the Care and Use of Laboratory Animals, National Research Council, 1996. The facility where this research was conducted is fully accredited by the Association for Assessment and Accreditation of

Laboratory Animal Care International. For immunizations, mice were randomly divided into groups of 10 and injected intraperitoneally with 10 μg of LASV VLP (Z+GPC or Z+GPC+NP) in 100 μL of sterile TNE. Ten mice were similarly injected with 100 μL TNE as vector control. One prime and two boosts were performed, three weeks apart, each with 10 μg of homologous LASV VLP. Mice were sacrificed by CO_2 _asphyxiation three weeks after the last boost and whole blood was collected by cardiac puncture. The plasma fraction was isolated and frozen at -80°C until analysis.

### IgG and IgM ELISA on recombinant LASV proteins and VLP

Murine immunoglobulin-γ endpoint titers to whole VLP, and IgG-γ to GP1 and GP2 were determined in serially diluted sera samples. Nunc MaxiSorp ELISA plates were coated with 2 μg/mL total VLP protein in carbonate buffer. Recombinant mammalian cell expressed LASV GP1 and GP2, produced by Vybion, Inc., Ithaca, NY, were coated on Nunc PolySorp ELISA strips, pre-blocked, and lyophilized by Corgenix Medical Corp., Broomfield, CO. Plates coated with VLP were blocked in 1X PBS, pH 7.4, 5% NFDM, 1% FBSΔ, 0.05% Tween-20, 0.01% thymerosal. The same buffer was used for all sera and secondary antibody dilutions. Mouse IgG was detected with a Horseradish Peroxidase (HRP)-labeled goat F(ab')_2 _anti-mouse IgG γ-specific reagent at 1:2500 dilution (KPL). Reactions were developed with TMB for 15 minutes at room temperature, stopped with 0.5 N H_2_SO_4_, and plates were read at 450 nm in a BioTek 808 ELISA reader. Viral antigen-specific IgG and IgM analysis in the sera of convalescent patients was similarly performed, with serum samples diluted 1:100 in NFDM blocking reagent, and detected with HRP labelled goat F(ab')_2 _anti-human IgG, γ or μ-specific reagents, respectively. Monoclonal antibodies to GP2 and NP were used as positive controls on antigen coated plates to verify presence of relevant epitopes on viral proteins. Total IgG fraction from naive mice was used as negative control antibody (ms IgG). Sera collected from North American volunteer blood donors that had never travelled to LHF endemic regions, and that were confirmed naive to LASV antigens by ELISA were used as negative controls. Serum from a patient that tested positive for NP-specific IgM and IgG antibodies in a recombinant NP ELISA was used as a positive control in these assays (G652-3).

### Electron microscopy

HEK-293T/17 cells were harvested at 72 hours post transfection with LASV gene constructs. Cells were pelleted by centrifugation at 200 ×g, washed once in cold (4°C) PBS, and fixed with 2.5% glutaraldehyde in phosphate buffer. Fixed cell pellets were embedded in 1% agarose prepared in phosphate buffer and allowed to solidify at 4°C. Cell pellets in agarose were post fixed with 1% osmium tetroxide, dehydrated in a graded series of ethanol, and embedded in epoxy resin. Thin sections were cut on a Leica UC6 ultramicrotome, stained with uranyl acetate and lead citrate, followed by examination on a Hitachi H-7100 transmission electron microscope.

### Statistical analysis and *in silico *tools

Statistical analysis of data was performed with GraphPad InStat, V3.06 (GraphPad Software, Inc., San Diego, CA), using Analysis of Variance (ANOVA), paired or unpaired Student's t test, and Pearson's correlation. The PeptideCutter analysis tool from the Swiss Institute of Bioinformatics ExPASy Proteomics Server was employed in the *in silico *analysis of predicted trypsin cleavage sites on LASV GP1 and GP2.

## Competing interests

LMB, FJG, and RFG are listed inventors, in addition to others, in a PCT application entitled "Soluble and Membrane-Anchored Forms of Lassa Virus Subunit Proteins", filed in April 2008. Additionally, LMB and RFG are listed inventors in a provisional application for United States letters patent entitled "Lassa virus particles and methods for production thereof", filed in September 2009. This work was performed as partial fulfilment of Ph.D. dissertation requirements for LMB. JNG, MLB, IJM, SAM, LAH, RJS, KAC, LEH declare no competing interests.

## Authors' contributions

LMB contributed to the experimental design, engineered the expression systems, performed data analysis, and drafted the manuscript. JNG generated LASV VLP, characterized morphological effects of VLP *in vitro*, performed VLP ELISAs with human sera, and helped draft the manuscript. FJG, MLB, and IJM developed the LASV IgG, IgM, antigen capture ELISA and performed assay optimization. SAM prepared and analyzed samples by electron microscopy. LAH manufactured recombinant proteins and provided critical review of the manuscript. RJS, KAC, and LEH contributed critical reagents and provided critical review of the manuscript. RFG contributed to the experimental design and provided critical review of the manuscript. All authors have read and approved the final manuscript.

## Supplementary Material

Additional file 1 **Graphic representations of recombinant constructs, mammalian plasmid vector, and single LASV gene expression**. A*i*. GPC gene with known domains (SP, signal peptide; GP1, glycoprotein 1; GP2, glycoprotein 2; TM, transmembrane; IC, intracellular; ER, endoplasmic reticulum retention signal). Signal peptidase (SPase) and subtilase SKI-1/S1P cleavage sites are indicated. Seven glycosylation sites on GP1 and 4 on GP2 are indicated by ***Y***. A*ii*. GPC construct with C-terminal FLAG. A*iii*. Nucleoprotein gene displaying putative helicase, RNA binding, WD40, repeated [R] domains, and pre-protein cleavage motif. A*iv*. NP with C-terminal 6X-HIS. A*v*. Z gene displaying myristoylation (myr), cyclin/CDK, nuclear receptor box (NR BOX), RING, and late PTAP and PPPY domains. A*vi*. Z gene with one glycine-6X-HIS domain inserted at amino acid position +3. A*vii*. Z gene with C-terminal 6X-HIS. B. Mammalian expression vector pcDNA3.1+_*int*A was used to generate all expression constructs outlined in these studies. C. LASV NP-3'HIS (lane 1), Z-3'HIS (lane 2), Z-5'glyHIS (lane 3), and GPC (lane 4) gene expression were analyzed by western blot. C*i*. Intracellular (C) expression of NP-3'HIS (60 kDa), Z-3'HIS (12 kDa), Z-5'glyHIS (15 kDa), and GPC (72 kDa). In the GPC lane, probed with an α-GP1 mAb, expression of monomeric GP1 was also detected (42 kDa). In culture supernatants (S), NP-3'HIS was not detected (C*ii*, lane 1). Z-3'HIS was present in supernatants at high levels (C*ii*, lane 2). Disrupting the myristoylation site on the N-terminus of Z prevented the release of the protein from cells (C*ii*, lane 3). The soluble GP1 component previously described through expression of GPC [11,12] was detected in supernatants (42 kDa) (C*ii*, lane 4). Click here for file

Additional file 2 **Transfection experiments with combinations of tagged and untagged Z, NP, and GPC constructs**. HEK-293T/17 cells were transfected in 6-well plates as outlined in Methods, with combinations of LASV gene constructs. VLP were purified through 20% sucrose cushions and subjected to western blot analysis. Blots were probed with αGP1, αGP2, αFLAG M2, αHIS mAbs, or αNP PAb. Lane designations: 1. Z; 2. Z-3'HIS; 3. Z+GPC+NP; 4. Z+GPC-FLAG+NP; 5. Z-3'HIS+GPC+NP; 6. Z-3'HIS+GPC-FLAG+NP; 7. Z+GPC; 8. Z-3'HIS+GPC; 9. Z+GPC-FLAG; 10. Z-3'HIS+GPC-FLAG; 11. Z+NP; 12. Z-3'HIS+NP. The Z-3'HIS+GPC+NP combination consistently generated the highest VLP yields with corresponding incorporation of all three LASV genes. Click here for file

Additional file 3 **DNA fragmentation and MTT cytotoxicity analysis of HEK-293T/17 cells transfected with LASV gene constructs**. A. Fragmentation assays were performed by resolving 2 μg of genomic DNA from transfected and untransfected cells on agarose gels. A low molecular weight DNA laddering effect consistent with apoptotic DNA fragmentation was not observed in any of the samples (n = 3). B. MTT cytotoxicity analysis of transfected cells, in 96-well format (n = 3). Vector only (pcDNA3.1+:intA), NP, GPC, and GPC-FLAG transfected cells did not display significant cytotoxicity when compared to untransfected controls (293T/17 cell ctrl) [p > 0.05]. Conversely, inclusion of the Z matrix gene, in native (Z) or 3'HIS-tagged format (Z-3'HIS), alone or in combination with any version of LASV GPC and/or NP resulted in significant reduction in MTT incorporation levels [p < 0.05 to p < 0.001, n = 3]. The numbered gel lanes in A. correspond to the bars in B. The p value for each transfection condition compared to the 293T/17 cell control is shown above the corresponding lane. Click here for file
