# Supplementary figures and images for: Lassa virus-like particles displaying all major immunological determinants as a vaccine candidate for Lassa hemorrhagic fever
Source: Virol J. 2010 Oct 20;7:279. doi: 10.1186/1743-422X-7-279 (PMC2984592; doi:10.1186/1743-422X-7-279)

## Slide 1
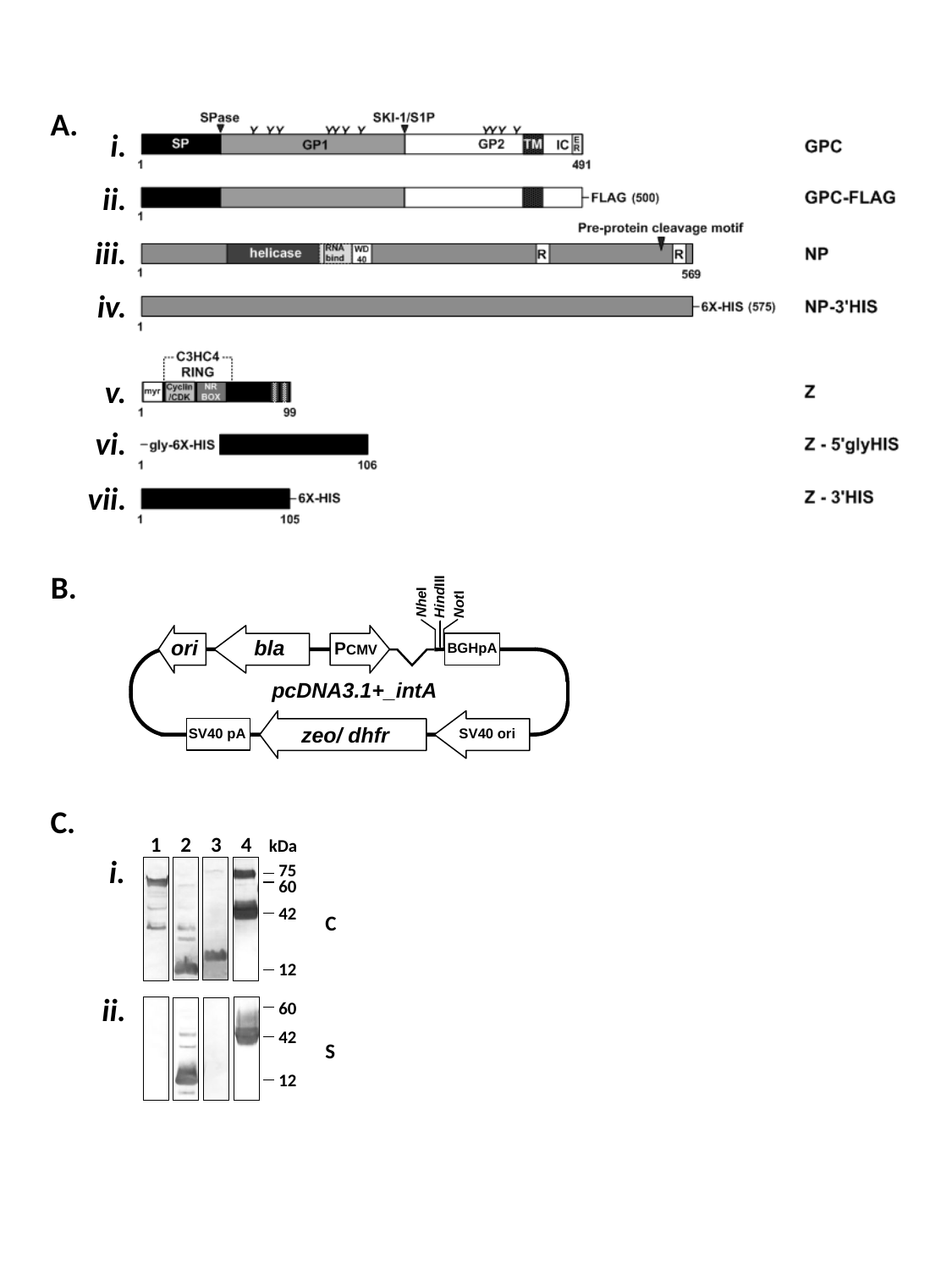

A.
i.
ii.
iii.
iv.
v.
vi.
vii.
B.
C.
1 2 3 4
kDa
i.
75
60
42
C
12
ii.
60
42
S
12

Supplement: Additional file 1 — Graphic representations of recombinant constructs, mammalian plasmid vector, and single LASV gene expression. Ai. GPC gene with known domains (SP, signal peptide; GP1, glycoprotein 1; GP2, glycoprotein 2; TM, transmembrane; IC, intracellular; ER, endoplasmic reticulum retention signal). Signal peptidase (SPase) and subtilase SKI-1/S1P cleavage sites are indicated. Seven glycosylation sites on GP1 and 4 on GP2 are indicated by Y. Aii. GPC construct with C-terminal FLAG. Aiii. Nucleoprotein gene displaying putative helicase, RNA binding, WD40, repeated [R] domains, and pre-protein cleavage motif. Aiv. NP with C-terminal 6X-HIS. Av. Z gene displaying myristoylation (myr), cyclin/CDK, nuclear receptor box (NR BOX), RING, and late PTAP and PPPY domains. Avi. Z gene with one glycine-6X-HIS domain inserted at amino acid position +3. Avii. Z gene with C-terminal 6X-HIS. B. Mammalian expression vector pcDNA3.1+_intA was used to generate all expression constructs outlined in these studies. C. LASV NP-3'HIS (lane 1), Z-3'HIS (lane 2), Z-5'glyHIS (lane 3), and GPC (lane 4) gene expression were analyzed by western blot. Ci. Intracellular (C) expression of NP-3'HIS (60 kDa), Z-3'HIS (12 kDa), Z-5'glyHIS (15 kDa), and GPC (72 kDa). In the GPC lane, probed with an α-GP1 mAb, expression of monomeric GP1 was also detected (42 kDa). In culture supernatants (S), NP-3'HIS was not detected (Cii, lane 1). Z-3'HIS was present in supernatants at high levels (Cii, lane 2). Disrupting the myristoylation site on the N-terminus of Z prevented the release of the protein from cells (Cii, lane 3). The soluble GP1 component previously described through expression of GPC [11,12] was detected in supernatants (42 kDa) (Cii, lane 4). [file 1743-422X-7-279-S1.PPTX]

## Slide 1
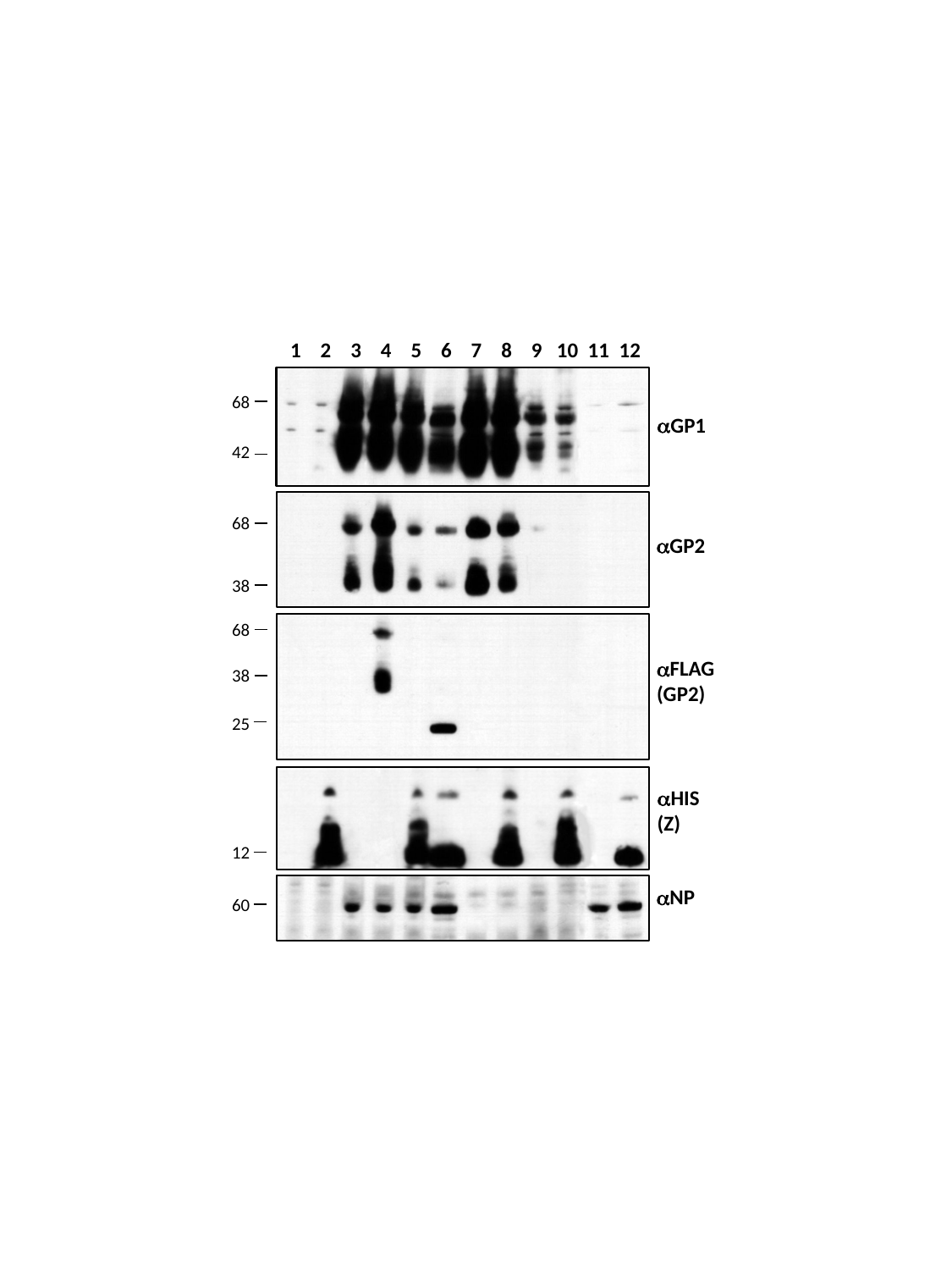

1 2 3 4 5 6 7 8 9 10 11 12
68
aGP1
42
68
aGP2
38
68
aFLAG
(GP2)
38
25
aHIS
(Z)
12
aNP
60

Supplement: Additional file 2 — Transfection experiments with combinations of tagged and untagged Z, NP, and GPC constructs. HEK-293T/17 cells were transfected in 6-well plates as outlined in Methods, with combinations of LASV gene constructs. VLP were purified through 20% sucrose cushions and subjected to western blot analysis. Blots were probed with αGP1, αGP2, αFLAG M2, αHIS mAbs, or αNP PAb. Lane designations: 1. Z; 2. Z-3'HIS; 3. Z+GPC+NP; 4. Z+GPC-FLAG+NP; 5. Z-3'HIS+GPC+NP; 6. Z-3'HIS+GPC-FLAG+NP; 7. Z+GPC; 8. Z-3'HIS+GPC; 9. Z+GPC-FLAG; 10. Z-3'HIS+GPC-FLAG; 11. Z+NP; 12. Z-3'HIS+NP. The Z-3'HIS+GPC+NP combination consistently generated the highest VLP yields with corresponding incorporation of all three LASV genes. [file 1743-422X-7-279-S2.PPTX]

## Slide 1
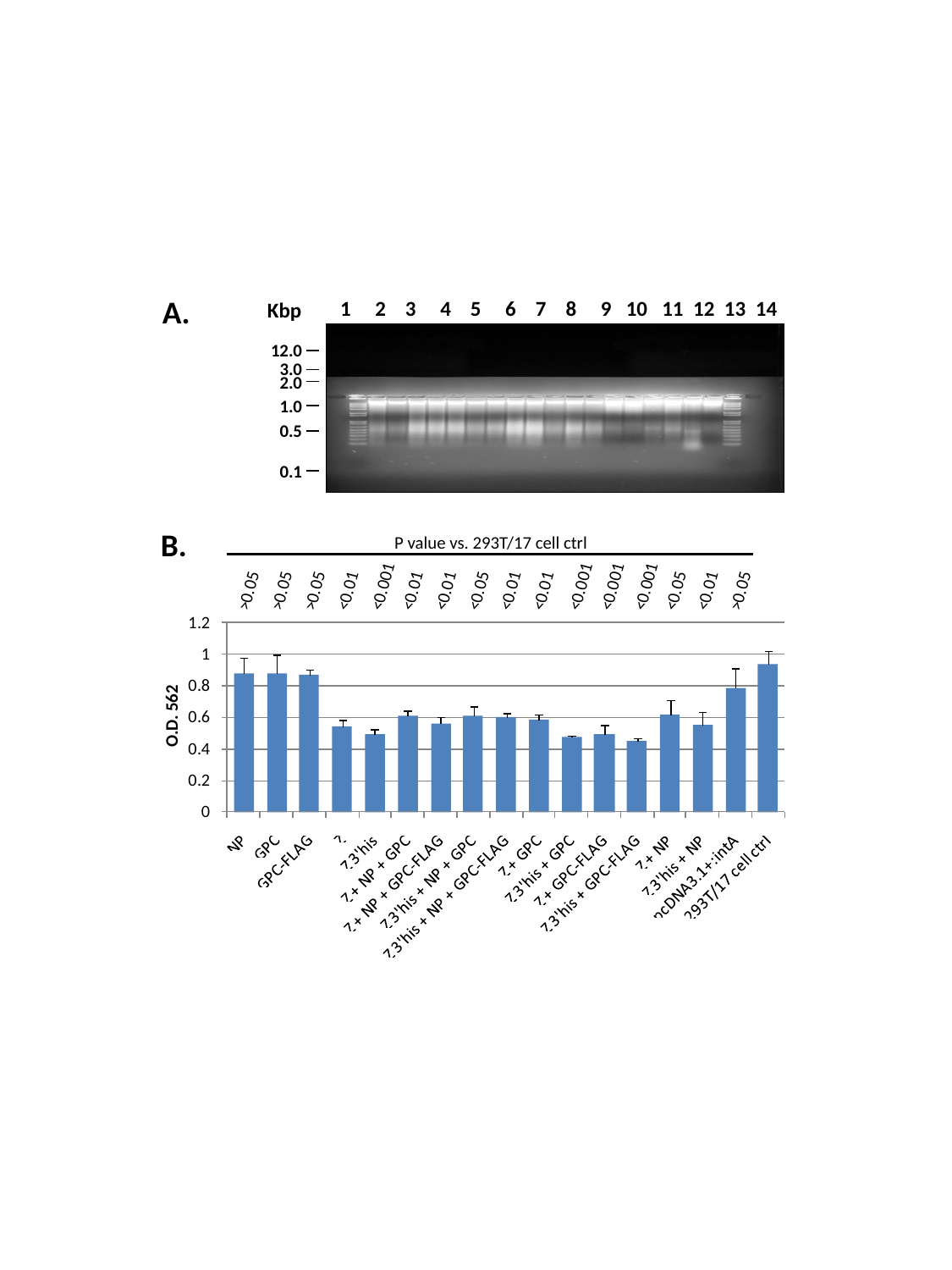

A.
1 2 3 4 5 6 7 8 9 10 11 12 13 14
Kbp
12.0
3.0
2.0
1.0
0.5
0.1
B.
P value vs. 293T/17 cell ctrl
<0.001
<0.001
<0.001
<0.001
>0.05
>0.05
>0.05
<0.01
<0.01
<0.01
<0.05
<0.01
<0.01
<0.05
<0.01
>0.05
O.D. 562

Supplement: Additional file 3 — DNA fragmentation and MTT cytotoxicity analysis of HEK-293T/17 cells transfected with LASV gene constructs. A. Fragmentation assays were performed by resolving 2 μg of genomic DNA from transfected and untransfected cells on agarose gels. A low molecular weight DNA laddering effect consistent with apoptotic DNA fragmentation was not observed in any of the samples (n = 3). B. MTT cytotoxicity analysis of transfected cells, in 96-well format (n = 3). Vector only (pcDNA3.1+:intA), NP, GPC, and GPC-FLAG transfected cells did not display significant cytotoxicity when compared to untransfected controls (293T/17 cell ctrl) [p > 0.05]. Conversely, inclusion of the Z matrix gene, in native (Z) or 3'HIS-tagged format (Z-3'HIS), alone or in combination with any version of LASV GPC and/or NP resulted in significant reduction in MTT incorporation levels [p < 0.05 to p < 0.001, n = 3]. The numbered gel lanes in A. correspond to the bars in B. The p value for each transfection condition compared to the 293T/17 cell control is shown above the corresponding lane. [file 1743-422X-7-279-S3.PPTX]
